# Exhaled Breath Analysis for Lung Cancer Detection Using Ion Mobility Spectrometry

**DOI:** 10.1371/journal.pone.0114555

**Published:** 2014-12-09

**Authors:** Hiroshi Handa, Ayano Usuba, Sasidhar Maddula, Jörg Ingo Baumbach, Masamichi Mineshita, Teruomi Miyazawa

**Affiliations:** 1 Division of Respiratory and Infectious Diseases, Department of Internal Medicine, St. Marianna University School of Medicine, Kawasaki-shi, Kanagawa, Japan; 2 B&S Analytik, BioMedicalCenter, Dortmund, Germany; 3 Reutlingen University, Faculty Applied Chemistry, Reutlingen, Germany; University of Chile, Chile

## Abstract

**Background:**

Conventional methods for lung cancer detection including computed tomography (CT) and bronchoscopy are expensive and invasive. Thus, there is still a need for an optimal lung cancer detection technique.

**Methods:**

The exhaled breath of 50 patients with lung cancer histologically proven by bronchoscopic biopsy samples (32 adenocarcinomas, 10 squamous cell carcinomas, 8 small cell carcinomas), were analyzed using ion mobility spectrometry (IMS) and compared with 39 healthy volunteers. As a secondary assessment, we compared adenocarcinoma patients with and without epidermal growth factor receptor (EGFR) mutation.

**Results:**

A decision tree algorithm could separate patients with lung cancer including adenocarcinoma, squamous cell carcinoma and small cell carcinoma. One hundred-fifteen separated volatile organic compound (VOC) peaks were analyzed. Peak-2 noted as n-Dodecane using the IMS database was able to separate values with a sensitivity of 70.0% and a specificity of 89.7%. Incorporating a decision tree algorithm starting with n-Dodecane, a sensitivity of 76% and specificity of 100% was achieved. Comparing VOC peaks between adenocarcinoma and healthy subjects, n-Dodecane was able to separate values with a sensitivity of 81.3% and a specificity of 89.7%. Fourteen patients positive for EGFR mutation displayed a significantly higher n-Dodecane than for the 14 patients negative for EGFR (p<0.01), with a sensitivity of 85.7% and a specificity of 78.6%.

**Conclusion:**

In this prospective study, VOC peak patterns using a decision tree algorithm were useful in the detection of lung cancer. Moreover, n-Dodecane analysis from adenocarcinoma patients might be useful to discriminate the EGFR mutation.

## Introduction

Recently the National Lung Screening Trial team reported that screening with low dose computed tomography (CT) reduced the mortality of lung cancer by about 20%. Low dose CT is an important screening test; however, it is expensive and there are risks associated with radiation exposure. On the other hand, breath analysis is easy-to-use and radiation-free. Gas chromatography and mass-spectrometry (GC/MS) [1]–[2] and chemical sensor matrices: quartz microbalance [3], surface acoustic wave [4], carbon-polymer array [5], colorimetric sensor [6], single-walled carbon nanotube [7] and gold nanoparticles [8], can detect volatile organic compounds (VOCs) in lung cancer from human breath. In addition, canine scent has focused on the diagnosis of lung cancer [9]–[10].

Ion mobility spectrometry (IMS) with multi-capillary column (MCC), a breath analysis device, can detect specific VOCs in patients with lung cancer [11]. IMS/MCC can detect a very low concentration of VOCs (normally in the ppbv- to pptv-range, pg/L to ng/L-range) in less than 8 minutes total analysis time and is superior to GC/MS as it can be applied at the bed-site and direct sampling can be taken without preparation [11]–[21]. In Europe, 550 MBq β-radiation sources are acceptable; however, for the Japanese market, regulations restrict ^63^Ni β-radiation sources to under 100 MBq. Therefore in this study, a 95 MBq ß-ionization source was used. The initial aim of this study is to confirm the reproducibility of IMS/MCC results (using BioScout: B&S Analytik, Dortmund, Germany) for a Japanese population.

Chemotherapy of lung cancer patients depends upon performance status, histological features, tumor staging, and molecular characteristics. Previously, 2 drugs combination chemotherapy including platinum has been performed as a first-line treatment for patients with advanced non-small cell lung cancer (NSCLC) considered as a single disease despite of its histologic and molecular heterogeneity. However, recently, the discovery of molecular abnormalities such as epidermal growth factor receptor (EGFR) mutation, and new agents such as EGFR tyrosine kinase inhibitor changed treatment of NSCLC. These led NSCLC treatment to the personalized therapy. Differences of histologic type and genetic alterations are the most important factors in decision of current lung cancer treatment. The second aim of this study is to confirm whether VOC patterns are able to detect histologically confirmed lung cancers, and driver mutations such as EGFR mutation.

## Methods

Breath analysis using an ion mobility spectrometer (IMS) was randomly performed in healthy volunteers and patients with lung cancer at St. Marianna University School of Medicine from 1 September 2011 to 14 January 2013. In all patients with lung cancer, breath samples were collected before bronchoscopy. The Ethics Committee of St. Marianna University School approved this study and written informed consent was obtained from all subjects (No1820). This study was registered with the University Hospital Medical Information Network Clinical Trial Registry (UMIN-CTR) (UMIN000006696, 000008328).

The exhaled breath of 50 patients (31 men, 19 women), with lung cancer confirmed histologically by bronchoscopic biopsy specimen was compared with 39 healthy volunteers (25 men, 14 women). Smoking histories of subjects were measured using pack-years.

### Ion mobility spectrometry (IMS)

IMS (BioScout, B&S Analytik, Dortmund, Germany) combined with a multi-capillary column (MCC, type OV-5, Multichrom Ltd, Novosibirsk, Russia) and coupled to a spirometer (Ganhorn Medizin Electronic, Niederlauer, Germany), as a CO2-controlled sample inlet unit was utilized. Table 1 shows the characteristics of ion mobility spectrometer.

**Table 1:** Characteristics of ion mobility spectrometer (BioScout).

Parameters	BioScout
Ionization source	^63^Ni (95 MBq)
Electric field strength	320 V/cm
Length of drift region	12 cm
Diameter of drift region	15 mm
Length of ionization chamber	15 mm
Shutter opening time	300 µs
Shutter impulse time	100 ms
Drift gas	Synthetic air
Drift gas flow	100 … 300 mL/min
Temperature	Room temperature
Pressure	101 kPa (ambient pressure)
MCC	OV-5, polar
Column temperature	40°C isotherm

The major parameters of breath analysis have been previously summarized [11]–[21] and will be discussed here in brief. IMS refers to the detection of ions formed from analysis at ambient pressure within a drift tube. The term ion mobility spectrometry refers to the method characterizing analysis in gases by their gas phase ion mobility. Normally, the drift time of ion swarms, formed using suitable ionization sources then passing through electrical shutters, are measured. Ion mobility for analysis can provide a means for detecting and identifying vapors. The drift velocity is related to the electric field strength by the mobility. Therefore, the mobility is proportional to the inverse drift time, which will be measured at a fixed drift length. IMS combines both high sensitivity and relatively low technical expenditure with a high-speed data acquisition. The time to acquire a single spectrum is in the range of 10 ms to 100 ms. Thus, IMS is an instrument suitable for process control, but due to the occurrence of ion-molecule reactions and relatively poor resolution of the species formed, it is generally not for identification of unknown compounds. Compared with mass spectrometry, the mean free path of the ions is much smaller as the dimensions of the instrument. An ion formed has a high number of collisions with carrier gas molecules on the drift way towards the Faraday-plate. However, because of the high vacuum conditions in mass spectrometry, an ion formed there will normally have no collision with other molecules during the drift. In the small time gap between the collisions the ion will gain energy from the external electric field and lose the energy by the next collision process. Consequently, a rather constant drift velocity will be reached. Therefore, an ion swarm drifting under such conditions experiences a separation process that is based on different drift velocities of ions with different masses or geometrical structures. Collection of these ions on a Faraday-plate delivers a time dependent signal corresponding to the mobility of the arriving ions. Such an ion mobility spectrum contains information on the nature of the different compounds present in the sample gas.

Compared to other analytical methods, IMS has a significantly large information density with comparative low burden in weight, power and size. Naturally, there are other analytical techniques, which contain much greater information density like mass spectrometry. Other techniques are smaller and more economical on power like surface acoustic wave sensors. IMS shows its specificity depending on ion size, chemistry and nature of the sample. It can be very high, through a combination of drift time and ionization properties. When it is always possible, hyphenated GC-IMS are preferred. By itself IMS is superior to MS and GC with respect to utilities, gas consumption, no vacuum is required and relatively low power requirements.

For spectrometry, a 95 MBq ^63^Ni ß-radiation source was applied for the ionization of carrier gas (synthetic air). Generally, the total number of ions formed is slightly lower using 95 MBq compared to 550 MBq. As a result, the total number of ions with the reactant ion peak in synthetic air will decrease the linear range marginally. For application cases like breath analysis mostly working on detection limits of analysis, the occurrence of analysis plays a more important role than the linear range. As shown later in this paper, the discrimination power and the detectability of the analyses in exhaled breath are not affected by the difference in the activity of the ionization source.

The spectrometer was connected to a polar MCC that functioned as a pre-separation unit. For MCC, the analyses of exhaled breath were sent through 1000 parallel capillaries, each with an inner diameter of 40 µm and a film thickness of 200 nm. The total diameter of the separation column was 3 mm.

The exhaled breath of subjects was taken directly through the spirometer using a standard mouthpiece containing an ultrasonic sensor without registering the 500 mL of dead volume on expiration. The contents of a 10 mL sample loop were added to the inlet of the MCC and transported to IMS, which was directly connected to the ionization region after pre-separation. The MCC and drift tube were held at 40°C. The carrier and drift gas used was synthetic air (Nippon Megacare, Tokyo, Japan).

### Statistical analysis

The peaks were characterized using Visual Now 2.2 software (B&S Analytik, Dortmund Germany) [14],[22]–[25]. All peaks found were characterized by their position with drift time (corresponding 1/K_0_-value) and retention time, and their concentration related to the peak height (table 1). Details of the data analysis procedure were realized based on the methods described in detail previously [15],[22]–[26] and summarized here [27]–[31].

For the different groups and each of the peaks, Box-and-Whisker plots were generated. The rank sum was provided by Wilcoxon-Mann-Whitney test using Bonferroni correction. Visual Now 2.2 was used to rank the peaks with the highest difference between groups.

The relation between the peaks found in BioScout and the analysis was realized by comparison using the Visual Now Version 110801 database (B&S Analytik, Dortmund, Germany), obtained by measurements described previously [11], [32]–[34]. In the present paper, peaks were correlated with the nearest analysis from the reference database and compared to the actual position of the peak.

## Results

All lung cancers were histologically proven by bronchoscopic biopsy samples. In 28 patients, transbronchial biopsy in peripheral pulmonary lesions using both endobronchial ultrasonography with guide-sheath and virtual bronchoscopic navigation was confirmed. In 22 patients, centrally located tracheobronchial lesions could be directly confirmed. The types of lung cancer were: 32 adenocarcinomas, 10 squamous cell carcinomas and 8 small cell carcinomas. Of 32 patients with adenocarcinoma, 14 were found to be positive for the EGFR mutation, 14 were negative for the EGFR mutation and 4 patients were positive for anaplastic lymphoma kinase (ALK) fusion. Lung cancer TNM staging showed: stage 1 = 13 patients, stage 2 = 6 patients, stage 3 = 8 patients and stage 4 = 23 patients. Seven of 39 healthy volunteers and 33 of 50 patients with lung cancer had smoking histories (table 2).

**Table 2:** Characteristics of patients.

	Healthy	Lung cancer
Sex		
Male	25	31
Female	14	19
Age	32±8	68±10
Pathological type		
adenocarcinoma		32
EGFR mutation (+)		14
EGFR mutation (−)		14
ALK fusion (+)		4
squamous cell carcinoma		10
small cell carcinoma		8
Tumor stage		
I (IA, IB)		13 (7, 6)
II (IIA, IIB)		6 (3, 3)
III (IIIA, IIIB)		8 (4, 4)
IV		23
Tumor Location		
Central		22
Peripheral		28
Smoking in pack-years	4.0±9.4	31.7±28.3

**Figure 1:**
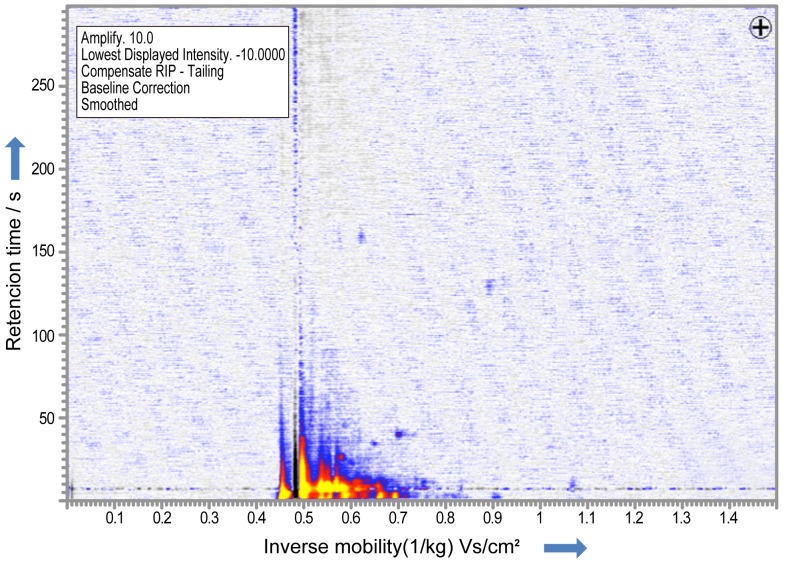
IMS chromatogram in a healthy volunteer. One hundred-fifteen VOC peaks were detected with ion mobility spectrometry in patients with lung cancer and healthy volunteers.

**Figure 2:**
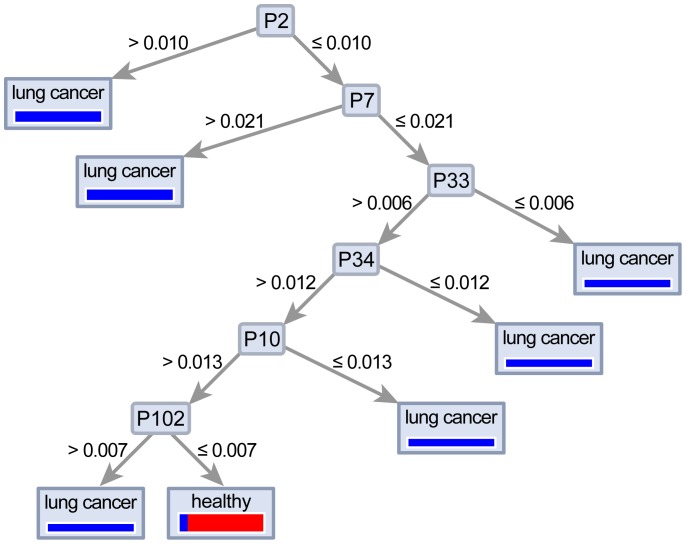
Decision tree algorithm to discriminate between healthy and lung cancer patients.

A total of 115 different peaks were compared with respect to the separation power in patients with lung cancer and healthy volunteers (Fig. 1). Ten VOC peaks were identified with significance higher than 95% (p<0.01) in patients with lung cancer. Of these, peak-2, which has the strongest VOC peak, is noted as the n-Dodecane using the IMS database and was able to separate values with a sensitivity of 70.0% and a specificity of 89.7%. The 9 other VOC peaks were also identified using the database (table 3). In addition, using a decision tree algorithm with n-Dodecane as starting point, a sensitivity of 76%, specificity of 100%, PPV of 100% and NPV of 76.4% were recorded (Fig. 2).

**Table 3:** Detection of VOC peaks using Visual Now database.

Peak	Description	1/K_0_	RT	P value
2	n-Dodecane	0.891	128.9	<0.001
6	3-Methy1-1 = Butanol	0.737	11.0	<0.001
11	2-Metylbutylacetat or 2-Hexanol	0.631	12.4	<0.001
22	Cyclohexanon	0.564	11.6	<0.01
23	Iso-propylamin	0.587	3.0	<0.01
37	n-Nonal or Cyclohexanon	0.716	10.4	<0.001
76	Ethylbenzol	0.564	9.8	<0.01
86	Hexanal	0.633	7.0	<0.01
109	Heptanal	0.671	13.6	<0.01
110	3-Methyl-1-butanol	0.608	14.0	<0.01

Lung cancer vs. healthy subjects.

**Figure 3:**
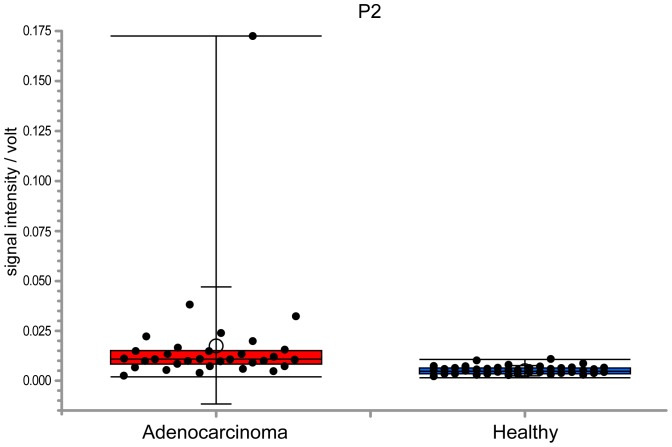
Box-and-whisker plots of peak-2 between healthy and lung adenocarcinoma patients. Peak 2 was significantly higher in patients with lung cancer (p<0.001). The box represents the 25th and 75th percentiles, the whiskers represent the range, and the lined box represents the median, whereas circles represent the mean. Lung adenocarcinoma patients revealed a significantly higher n-Dodecane VOC peak than healthy volunteers and the n-Dodecane VOC peak could separate values with a sensitivity of 81.3% and a specificity of 89.7%.

**Figure 4:**
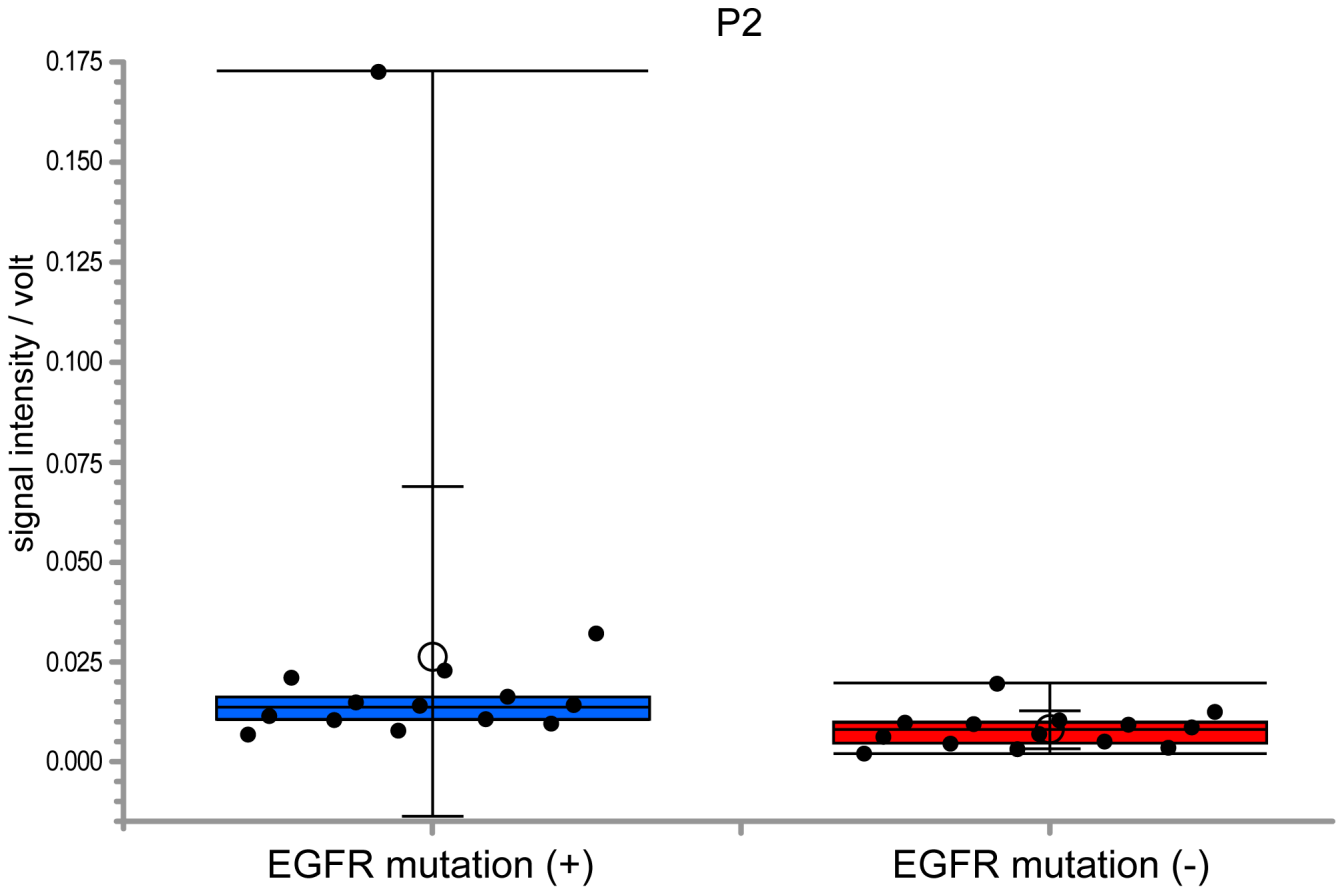
Box-and-whisker plots showing the IMS signal intensity of peak-2 in adenocarcinoma patients positive and negative for EGFR. Fourteen patients with EGFR mutation displayed a significantly higher n-Dodecane peak with a sensitivity of 85.7% and a specificity of 78.6% (p<0.01) than in 14 adenocarcinoma patients without the EGFR mutation.

**Figure 5:**
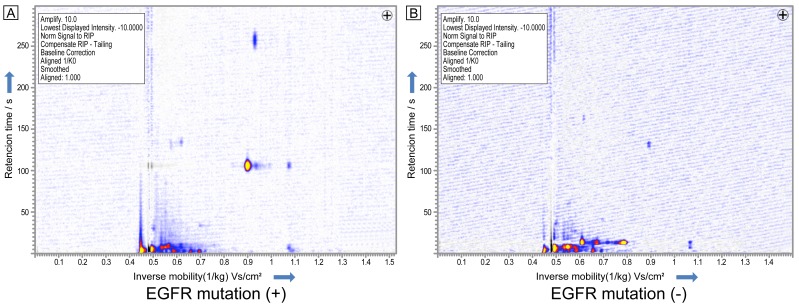
IMS chromatogram in patients with lung adenocarcinoma positive for EGFR mutation (A) and negative for EGFR mutation (B).

Comparing VOC peaks between adenocarcinoma and healthy subjects, 11 VOC peaks were found to have significance higher than 95% (p<0.01) and n-Dodecane (peak-2) was able to separate values with a sensitivity of 81.3% and a specificity of 89.7% (Fig. 3). In addition, 14 lung adenocarcinoma patients positive for EGFR mutation displayed a significantly higher n-Dodecane VOC peak than for 14 lung adenocarcinoma patients negative for the EGFR mutation without 4 patients positive for ALK fusion (p<0.01), with a sensitivity of 85.7% and a specificity of 78.6% (Figs. 4 and 5).

**Figure 6:**
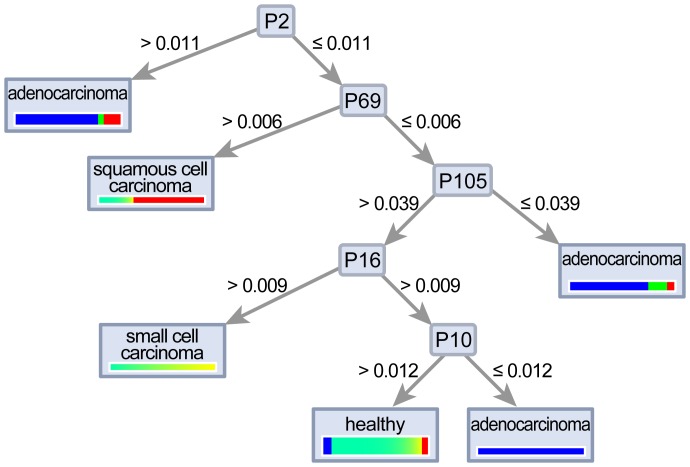
A decision tree algorithm could separate small cell carcinoma, squamous cell carcinoma and adenocarcinoma.

Comparing VOC peaks between squamous cell carcinoma and the healthy group, 11 VOC peaks were found to have significance higher than 95% and peak-69 was able to separate the best value with a sensitivity of 97.4 and a specificity of 60.0% (p<0.001). Comparing VOC peaks between small cell carcinoma and healthy subjects, peak-6 was found to be significantly higher than 95% (p<0.01) with a sensitivity of 97.4% and a specificity of 50.0%. In addition, a decision tree algorithm could separate histological types of lung cancer and healthy volunteers (Fig. 6).

## Discussion

In this prospective study, VOC peak patterns using a decision tree algorithm were useful in the detection of lung cancer. We found that some VOC peaks displayed significant differences between patients with adenocarcinoma, squamous cell carcinoma, small cell carcinoma and healthy volunteers. In addition, some VOC peaks positive for the EGFR mutation displayed significant increases, especially the n-Dodecane peak, which was the most valuable biomarker. VOC analysis using IMS is expected to be an important detection test for lung cancer. To our knowledge, this is the first study to show that n-Dodecane analysis from adenocarcinoma patients might be useful to discriminate for the EGFR mutation.

VOC analysis of lung cancer using GC/MS has been used extensively since 1985. In GC/MS, some VOC models were used to analyze significance, with a sensitivity and specificity of 54 to 100% and 67 to 100%, respectively [35]. Westhoff et al. was the first to report VOC analysis for lung cancer using IMS. He reported that 23 VOC peaks from exhaled breath could separate lung cancer and a healthy control, unaffected by smoking history [11]. However, spectrometry technologies using breath sampling were affected by ambient conditions, oral odor and nutrition. Direct airway sampling under bronchoscopy was negligible for oral odor and some VOC peaks displayed significant differences between the lung tumor site and the normal site. Moreover, some VOC peaks, 2-Butanol, 2-Methylfuran and n-Nonanal, proved useful to separate adenocarcinoma and squamous cell carcinoma [36]–[37]. For lung adenocarcinoma, n-Dodecane was found to be an important VOC peak for both breath analysis and bronchoscopic sampling and was reported to be associated to patients with lung cancer [36]–[37].

It is known that East Asian NSCLC patients have higher instances of EGFR mutation [38]–[40]. Driver mutations, including EGFR, have focused on lung cancer and other malignant tumors [41]–[43]. The EGFR mutation has a higher instance than other driver mutations in lung cancer and is sensitive to the EGFR tyrosine kinase inhibitor. The results of this study show that lung adenocarcinoma positive for the EGFR mutation tends to increase the intensity of some VOC peaks using IMS. EGFR may have a specific metabolism that may produce various VOCs. The detection of EGFR mutation needs surgical specimen, bronchoscopic or CT-guided needle biopsy tissue, bronchial lavage fluid and pleural effusion with tumor cell. A previous study reported exhaled breath condensate could evaluate EGFR mutation. However it was still difficult to detect EGFR mutations in exhaled breath condensate because cellular components presented in exhaled breath condensate are not representative of the tumor [44]–[45]. The analysis of VOC patterns including a decision tree algorithm may be useful to detect EGFR mutation emitted from lung cancer cell lines in the future.

This study had some limitations. First, the sample size was small and larger sample studies are required. Although more patient breath samples are needed to overcome potential problems with statistical investigations, in previous literature sample sizes for breath analysis had been smaller when compared to the present study [36]–[37]. Beside the major question to have more breath samples of patients than peaks to overcome potential problems with statistical investigations in general, here 89 samples were investigated and 115 peaks were found. Second, VOCs in patients with lung cancer may be affected by smoking history. It should be noted, if the differences were not related to tobacco smoking in lung cancer patients, which was considered in detail by Westhoff et al. [11] showing, that in both groups including a higher number of smokers and non-smokers the differentiation using ion mobility spectrometry was successful. For the molecules investigated by IMS in this study, the differences were independent of smoking status and significant for both groups. In the study of Westhoff et al. [11]. there was no database available to identify the analysis. Recently, Darwiche et al. [36] showed by comparison of measurements taking samples of air from the same patient at the cancer site and non-cancer site during bronchoscopy, differences found were related to the place the sample was taken, directly over cancer cells or on the other lung site. Third, in accordance with Japanese regulations, restrictions of ^63^Ni β-radiation sources of under 100 MBq have been set for this Japanese pilot study, which is lower than European restrictions. However, the current study results show that IMS with a 95 MBq β-radiation source could discriminate between healthy volunteers and patients with lung cancer successfully. Therefore, creating a database for the Asian population in relation to VOC peaks and substances may be required. In future studies, multi-center trials using IMS are needed to analyze lung cancer.
